# Analysis of the concept of informal economy through 102 definitions: legality or necessity

**DOI:** 10.12688/openreseurope.13990.1

**Published:** 2021-11-04

**Authors:** Arturo Luque

**Affiliations:** 1 Business Administration, Universidad del Rosario, Bogotá, 111711, Colombia; 2 Social Sciences and Law, Universidad Técnica de Manabí, Portoviejo, Manabí, 130105, Ecuador

**Keywords:** informal economy, ethics, legality, social economy, Sustainable Development Goals

## Abstract

The processes of informal economy are well established, but the same cannot be said of their conceptual treatment in the academic literature. They constitute complex phenomena that cut across sectors and disciplines and give rise to other elements that simultaneously reject and encourage them. For many formal stakeholders in the economy, they are an enemy to be beaten; for the authorities, informal activity is seen as a loss of revenue for the state coffers; for the Sustainable Development Goals, by implicitly recognizing them in goal 8, they constitute a paradigm shift.  Meanwhile, the reality for those involved in the informal economy is that it is a way of life and not a mere choice, one that leads to the most social of all economies: that of necessity. There is no consensus among academics on informality and its ramifications, hence the need to analyze the processes of informal economy from its theoretical construction with the purpose of discovering its range and depth, as well as its interrelationships and theoretical implications. To achieve this, 102 definitions of informal economy were analyzed by identifying and deconstructing their dimensions and performing a frequency count of their citation in Google Scholar. This analysis demonstrated the lack of cultural elements in the definitions, which are the true underlying cause of the phenomenon, and the over-prominence afforded to legal dimensions.

## Introduction

The world progresses, but its principal problems of poverty and inequality remain. Much of globalized society is engrossed in what they see as the pressing reality of climate change, derived from the exhaustion and mistreatment of the earth, and exacerbated by the unprecedented consumption of goods and services. Meanwhile, another part lives in an abyss of labor exploitation that is either overt and unabashed, or disguised through the legal processes of outsourcing, relocation, and international fragmentation of the value chain. Corruption and economic domination have produced a new *de facto* social class in developed countries: the precariat ( Guamán & Luque, 2019; Luque *et al.*, 2016; Standing, 2013). A great part of the world’s citizens finds themselves in this chasm, having been forced out of their natural ecosystem ( Sassen, 2015); in this abyss, any means of survival is valid including the processes of the informal economy. These comprise more than 90% of micro and small enterprises (MYPE) globally ( International Labour Organization [ILO], 2019) as well as a significant part of the rural economy in developing countries ( ILO, 2020b). In fact, the informal economy accounts for 10–20% of GDP in well-established economies and up to 60% in emerging economies ( Schneider & Enste, 2002), and two billion people worldwide are employed informally, which represents more than 60% of the global workforce. 93% of all informal employment is in emerging and developing countries: in Africa, 85.8% of jobs are informal; the proportion is 68.2% in Asia and the Pacific, 68.6% in the Arab States, 40.0% in the Americas, and 25.1% in Europe and Central Asia ( ILO, 2018). In Latin America and the Caribbean, about 140 million workers are in a situation of informality; according to Salazar-Xirinachs and Chacaltana (2018, p.15):

“These are workers who are not covered by labor law and are not, therefore, subject to the relevant formal regulations of the world of work or employment justice; they make no social security contributions and, consequently, do not participate in the welfare state or the social contract that characterizes modern societies; they mostly have low-productivity jobs and, as a result, their incomes are low. At worst, many of them live in poverty or extreme poverty; and at best, they comprise what analysts call "vulnerable groups”.

In response to this panorama, the member states of the United Nations agreed on the creation of the 2030 Agenda ( https://sdgs.un.org/2030agenda), which covers the three dimensions of sustainability (the economic, the social, and the environmental), and includes 17 Sustainable Development Goals (SDGs) ( https://sdgs.un.org/goals) The eighth of these SDGs has the aim of promoting sustained, inclusive, and sustainable economic growth, full and productive employment and decent work for all. It is estimated that more than 600 million new jobs will have to be created by 2030 bearing in mind that 780 million people do not currently earn enough to keep them above the poverty line of $2 per day ( ILO, 2020a). Consequently, the International Labour Organization (ILO) recommendation No. 204 on the transition from the informal to the formal economy ( ILO, 2015) mandates that the creation and sustainability of enterprises and decent jobs be facilitated and promoted, and steps be taken to prevent the informalization of employment.

Given the scale of the issue and the potential remedies being sought by international bodies, it is necessary to analyze the factors that contribute to the development of processes of informality in order to better define the dimensions involved. This study is based on a conceptual analysis with the specific aim of clarifying whether informality is concerned only with economic aspects or whether conditions of greater depth or interconnectedness are involved (see Table 1; Luque, 2021). These conditions are often overlooked in definitions of informality but can have an important role to play.

**Table 1.** Definition coding for each dimension with full search chain.

Dimensions, coding and related search of informality processes		
Dimension	Definition coding	Search chain
Economic	Economic or financial aspects that affect and promote informality processes	‘economic inclusion’
		‘weakening of control of regulatory bodies'
		‘protection of economic rights over human rights’
		‘equity of tax payments for big business’
Legal	Established relationships of regulatory nature that cause asymmetries	‘globalization processes’
		‘trade union freedom’
		‘regulatory and monitoring bodies for global action’
		‘new forms of partnership and association'
Social	Relationships and impact of the development of informal activity on society	‘formal recognition of social economy processes'
		‘quality of life’
		‘welfare state’
		‘processes of fair treatment’’
		‘alternative modes of life'
Political	Legal regulation and the absence of it based on political ideals/ programs	‘free trade and investment agreements with instruments for the resolution of private conflicts'
		‘lack of rights and employment deregulation'
		‘precariousness of employment’
		‘promotion and conservation of ancestral traditions'
Cultural	Ensemble of material and spiritual assets such as language, lifestyles, customs, traditions, habits, values, patterns, tools and knowledge	‘collective identity’
		‘undefined aspects that constitute society’
		‘worldview’
		‘cooperativism’
		‘solidarity’
		‘value of heritage’
Ethical	Principles based on ethics, customs, and morals ^1^	‘continual cost reduction’
		‘investments in countries in a state of conflict'
		‘corporate corruption’
Labor	De-structured view of the development process	‘fairer, more ethical and inclusive growth'
		‘fair trade’
		‘precariousness of employment’

Source: compiled by the author.

### The processes of informality and the development of its terminology

The term ‘informal sector’ was initially coined in 1972 in an ILO employment advisory mission to Kenya; from there it developed to ‘informal economy’, making it clear that informality is not limited to a ‘sector’, but is an alternative way of conducting almost all economic activity ( ILO, 2020c). The basic definition of informality includes elements of conflict and cooperation ( Thompson & Smith, 2009) as well as compatibility with the processes of marginality and economic domination ( Castells & Portes, 1989a; Harvey, 2007). Informality is measurable ( ILO, 2013), but it involves multiple heterogeneous causes, situations, and phenomena of great magnitude and depth, extending to aspects of self-employment ( Slavnic, 2010). However, it is possible to distinguish between the origin and the nature of informality: Mars (1982) approached the subject extensively from the point of view of why workers seek illicit remuneration for carrying out their activities. The processes of informality are imbued with strong cultural components characterized in turn by common denominators such as the lack of decent employment or the conditions that provide it (although, in certain cases, informal workers receive better wages than permanent, formal workers ( Chen *et al.*, 2005)). 

According to de soto (1989, p.11), informality is technically an illegal activity, albeit one that lacks "antisocial intent". From a business perspective, the impact of this is limited ( Khavul *et al*., 2009; Siqueira & Bruton, 2010) notwithstanding the doubts expressed by many academics and experts in management and entrepreneurship ( Ram *et al*., 2017). In fact, there may be informal, unregistered enterprises that, through their activity, receive revenue from the legal production of goods and services ( Bruton *et al*., 2012, p 1). In the view of the Inter-American Centre for Knowledge Development in Vocational Training (ILO/CINTERFOR), informal work includes:

“All paid work (e.g., both self-employment and wage labor) that is not registered, regulated or protected by legal or regulatory frameworks, as well as unpaid work undertaken in a for-profit business. Informal workers do not have stable contracts of employment, employee benefits, social protection or worker representation.” ( ILO/CINTERFOR, 2020b)

Among the characteristics of informal work is a lack of definition of the place in which the activity takes place, a lack of schedules and working hours, deficient means and resources, and irregularity of income; additionally, it is often carried out in an insecure and unhealthy environment without any kind of employment or social protection. Payment for the production of goods and services is typically unregistered in order to evade taxation, social security payments, and to avoid legal requirements ( Williams & Windebank, 1998, p. 4).

The causes of informality are many, but the economic context in a country is the decisive factor, followed by the legal situation, political stability, and certain microeconomic circumstances such as poor education, discrimination of all kinds (including that which is legally sanctioned), and various aspects of deprivation that lead to limited access to decent housing (both for rent and property). This is compounded by lack of access to financial services and, in turn, to markets (despite the constant rise of the cooperative sector, which offers savings and credit). Many studies show that the burdens associated with tax and social welfare systems are the key drivers of the growth of the informal economy ( Alm *et al*., 2006; Dell'Anno, 2003; Tanzi, 1980). Indeed, widespread and inflexible regulations also push an increasing number of workers and companies to join the informal sector ( Hoa, 2019). This over-zealous regulation combines with the processes of economic recession to favor models of disruptive economy. Companies such as Uber ( https://www.uber.com), Glovo ( https://glovoapp.com/), Cabify ( https://cabify.com), Airbnb ( https://www.airbnb.co.uk/), OpenTable ( https://www.opentable.co.uk/), Yelp ( https://www.yelp.com), Deliveroo ( https://deliveroo.com), and the giants, Amazon ( https://www.amazon.com) or AliExpress ( https://www.aliexpress.com), inherently offer little social coverage, taking advantage of the cracks in labor law deregulation to empower themselves and overrun the economic system, having forced out the traditional companies in their sector.

### Poverty as an element of disruption and domination

Poverty is not incompatible with richness of spirit. Nonetheless, economic deprivation conditions much of human existence, above all, quality of life and the prospects of the next generation. Poverty has detrimental consequences even for the lives of the rich: a society in which greater equality is achieved on the basis of the processes of true equity and a state that implements strong social policies will tend to be more peaceful and settled and obtain greater cost savings ( Wilkinson & Pickett, 2011). However, certain companies exploit the model of necessity, which consists in eroding any type of social assistance (the welfare state) and using the processes of corporate social responsibility to disguise the deficiencies of the care system. Such models rely entirely on goodwill but lack any enforceability ( Ruggie, 2001). The situation is aggravated by the ability of states to displace their policy commitments and social responsibility obligations with the blessing of supranational organizations ( Luque & Álvarez, 2021; Mason, 2016; Passet, 2013). At the same time, processes of corruption are occasionally sponsored by (usually transnational) companies in order to undermine the labor market, while monopsony stretches the very fabric of small and medium-sized enterprises ( Luque, 2018). It should be noted that many developing economies and models in transition demonstrate that informal economies of considerable size can coexist with the expansion of the formal economy and achieve good growth results ( ILO, 2014, p. 9).

“Many factories repeatedly issued unlawful short-term contracts to avoid paying workers maternity and other benefits, and to intimidate and control them. Small factories that subcontract to larger export-oriented factories are more likely to hire workers on a casual basis, making it harder for workers to assert their rights because they risk being easily fired. Apparel brands have not taken adequate steps to end the illegal short-term contracts in their supplier factories–even where their supplier codes of conduct have clauses limiting their use.” ( Human Rights Watch, 2015)

Faced with this situation, much of the public, committed to preserving their quality of life, look for easy credit (often accepting usurious interests) to the extent that uncontrolled debt becomes a social leitmotif. Many governments mirror this credit debt on a global scale ( World Bank, 2020). In developed countries, gambling has become widespread, and bookmakers have multiplied, encouraged by the state, whether as a kind of social anesthesia or as a squalid source of tax revenue ( Humphreys *et al*., 2020; Lopez-Gonzalez *et al*., 2019). According to Francisco and Bergoglio "The culture of relativism is the same pathology that makes one person take advantage of another and treat them as a mere object, pushing them into forced labor, or making them a slave to debt" (2015, p. 95). From new forms of poverty emerge new classes of the poor: the victims of climate, politics, economics and hunger, they range from refugees to academics ( Oliván & Luque, 2019). According to Beck:

“The rich, who coincidentally are the most resourceful and powerful actors on the political scene, do not need the poor even for the salvation of their souls (which, in any case, they do not believe they have and whose care they would not consider worthy of their interest), nor even to continue being rich, nor yet to grow even richer (which they believe would be easier if there were no need to share a portion of their wealth among the poor).” ( Beck, 1998, p.90).

## Methods

**Figure 1.**
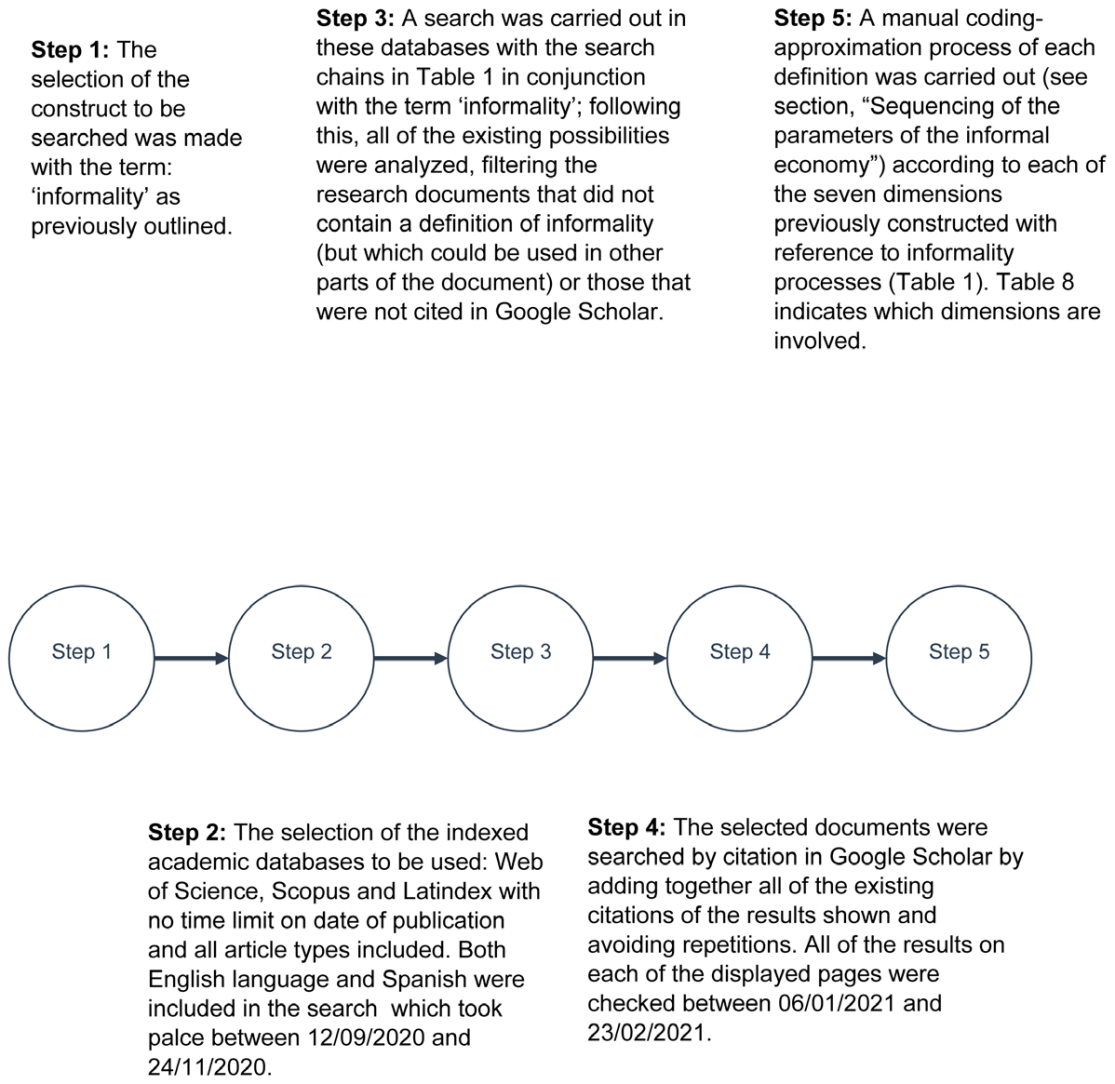
Flowchart of the search method used in this study within inclusion criteria. Source: compiled by the author. See Luque (2021).

The work presented here is not intended as an encyclopedic treatise but is rather an attempt to bring together a wide variety of methodological approaches that reflect both the conceptual evolution and the cross-disciplinary elements that comprise the study of the informal economy. The constructs themselves include aspects far removed from the needs or existing realities of the phenomenon, making them complex to understand ( Han, 2020). The study undertook an analysis of reasoned theoretical approaches that contain epistemological problems, which highlights the difficulty of providing generalized solutions to the many questions and dilemmas that surround this topic ( Fourez, 1994). The study consisted of three steps: 1) A search was carried out for definitions of the informal economy and related constructs through the existing literature using indexed databases, which in turn were part of Google Scholar ( https://scholar.google.com/). The selection of the indexed academic databases to be used were: Web of Science, Scopus and Latindex with no time limit on date of publication or type of paper. Both English language and Spanish publications were included in the search between 12/09/2020 and 24/11/2020. 102 definitions were found by this method between 12/09/2020 and 24/11/2020 (see Table 8; Luque, 2021). Figure 1 provides a summary of the steps carried out in the process of definition selection with inclusion criteria and subsequent categorization. From this, a large group of 102 linked units of analysis (UA) was obtained, which was used to define the concept under study (see Table 8; Luque, 2021) and to convert verbal or visual data into numerical data for the purposes of analysis ( Bourque, 2004).

 2) Seven dimensions were identified through content analysis by applying a manual coding-approximation process, from which the general description and, subsequently, the scope of each dimension were obtained as outlined below. 3) A frequency count in Google Scholar of all definitions was performed, measuring the impact through citations to then calculate the relative use of each dimension. It is necessary to understand that the relationship of the identified constructs is not of equal significance, the most widely used having a greater impact ( Donahue *et al*., 2015; Howes & Solomon, 1951; Kageura & Umino, 1996; Luque & Herrero-García, 2019; Murphy, 1992).

The relative usage of each definition can be obtained by comparing the frequency counts in an internet search engine. It is empirically evident that the internet provides frequency counts that are of great validity compared to other linguistic databases. Google Scholar, an engine specialized in scientific-academic searches that orders results by relevance, is one of the most effective free-access academic search engines globally.

### Establishing the dimensions and manual coding scheme in informality processes

The definitions were gathered through an extensive literature review, which consisted of journal articles, technical reports, and web pages. Dimension selection and classification was undertaken keeping in mind the depth, scope, and interconnectivity present in informal economic processes ( Luque & Herrero-García, 2019). This was achieved through the following procedure: a) The asic ideas to be used in the structuring of informality processes were defined, which in turn would serve to guide the research design; b) Once all of the definitions and approximations had been read, their depth and implications were observed and noted ( Bulmer, 1979; Nosofsky, 1986); c) From there, a theoretical framework was built for each dimension, justified by their treatment in the existing academic literature: in this case, seven were required. The same systematization can be replicated for other research, adapting the number of dimensions in relation to the existing literature that justifies them; d) Each definition of informality was analyzed at a conceptual level to discover whether it contained the preconfigured implications in each one of the dimensions; if so, they were included in Table 8 for subsequent counting and analysis. Initially, the MAXQDA ( https://www.maxqda.com/) program was to be used to carry out a semantic analysis, but eventually an iterative study carried out manually by the author was preferred due to the interpretive complexity of each informality structure identified. This procedure resulted in a grouping of related concepts through seven dimensions: economic (Eco), legal (Leg), social (Soc), political (Pol), cultural (Cul), ethical (Eth), and labor (Lab) ( Table 1; Luque, 2021) ( Rahman *et al*., 2019; Torugsa *et al*., 2013; Visser, 2006).

The economic dimension is directly related to the living conditions of people engaged in informal activities and, therefore, to informal economic processes. It must be made reasonably easy for all public-private actors to introduce more equitable and progressive aspects at the fiscal and legal level, and to articulate direct relationships between all types of companies and the immediate environment in which goods are produced or services are managed. The situation must also be open to regulation by understanding enterprises to be one more living element of the community rather than immovable and sacrosanct institutions.

The legal dimension considers the development and establishment of legislative and regulatory measures, especially those that have a bearing on the general interest. Among the most important are the processes of liberalization, with clear benefits for the ruling classes, such as: deregulation of markets; collusion of supranational bodies; lack of competition; protection of monopolies; creation of bespoke legislation; veiling of the activities of lobbies; caps on democratic processes; privatizations of profitable public enterprises; tax reforms in favor of the prevailing economic model; protection of economic rights over and above human rights. To these can be added regulatory asymmetries in free trade agreements with inadequate means of redress or democratic control, the imposition of private arbitration tribunals that undermine domestic jurisdiction as a means of dominating nation states, and the imposition of tax-containment processes tailored to the needs of transnational corporations ( Luque & de Pablos, 2016). The social dimension has a clear relationship with the processes of common welfare. There is an overlap of interests and relationships between individuals and life in society since individuals need others to fulfill their requirements. Therefore, it is necessary to develop all available tools, both those that examine the endogenous factors inherent to continuous learning processes, and those of an exogenous nature derived from the environment and based on continuous feedback; both have quality of life as their common denominator. Society responds to threats and aggressions of all kinds, such as the processes of exclusion and expulsion, gentrification, and economic domination; the informal economy is a clear expression of a social response to these abuses that is both rapid and proportionate. The political dimension is based on the social organization of coexistence. Both individuals and their environment are placed at the center of the social construction and form its nucleus. The interests of any society share common elements such as beliefs and values but also dissonant aspects such as the organizational model, processes of exclusion (by act or omission), or priorities implicit in government interventions. Within this context, civil society articulates its mechanisms of action, including civil disobedience as legitimate protest; these include unlicensed selling, peaceful resistance or the adoption of policies within the regulated social economy. Indeed, these policies seek to address real needs rather than maximize the economic benefit of the democratic majority. The ruling classes tend to regard informal economic processes as a source of illicit income and a feature of those who abuse their citizenship status, even to the extent of seeing them as an anti-system element, yet the great majority carry out informal activities as a form of subsistence to avoid poverty or as a response to exclusion. The cultural dimension is not confined to intellectual aspects and also not reserved for the economic elite or well-off. Rather, it is a construct of accessible elements in continuous motion. Culture is not always clearly defined, but it constitutes all kinds of expressions and is a clear element of vitality in society, a predictor of freedom and development. It may be true that the informal economy has an element of the anti-establishment, but informality as a recourse to covering basic needs is a clear example of the strength of a democracy. The ethical dimension is a permeable membrane between effective decision-making by citizens involved in the informal economy and their practical consequences. There is clear freedom of action when it comes to choosing what to produce, how to produce it, and how it will be distributed with a clear social commitment. This is based less on accumulative processes of wealth and more on the common good. However, three underlying limitations emerge: 1) The personal, based on less avaricious imperatives that are limited by the legal system, the family, and environment; 2) The organizational, derived from corporate and public policies exempt from limitations or control and enjoying regulations tailored to their interests; 3) The social, in which many of the mechanisms for the detection and containment of systemic problems are ineffective. These limitations persist despite the knowledge of such pathologies within a society that, recognizing its own deficiencies, is lacking in the tools for their remedy ( Bauman, 1999). The labor dimension not only analyzes the labor market or its characteristics, but the qualitative aspects of conditions for carrying out work for which legitimate access is denied by regulations. This dimension departs from the structuralist vision of development, taking an inclusive view of all kinds of activities that generate value within an ethical framework. Measures should be adopted to bring informal activities within the fold of the common good and in line with existing circumstances such as family and the ancestral traditions and practices of ethnic groups. To highlight this point, Italy's former ambassador to India and Iran, Roberto Toscano, when asked about labor protection systems, noted, "it seems clear to me that trade unionism has been defeated in the most developed world, and hardly exists in the least developed: in India 90 percent of workers are "informal" (no employment contract, no rights, no trade unions)" ( Luque *et al*., 2016, p.12).

### Sequencing of the parameters of the informal economy

According to the procedure described by Luque & Herrero-García (2019), the initial bibliographic search tentatively selected a series of constructs related to the informal economy and processes of informality as formulated by academics and/or experts on the subject through his already published indexed manuscripts (see Methods and Figure 1). Boolean searches for keywords, such as those set out in Table 1, were introduced, forming the basis of the combinatorial sets and the logic for the searches in Google Scholar of linked databases (Scopus, Web of Science, and Latindex) between 12/09/2020 and 24/11/2020. Basic operators such as AND, OR and NOT connect the keywords for the searches to narrow down or expand the results.

In this context, the total UAs were manually categorized in an iterative sequence according to the conceptual fit of each UA in each of the seven previously formed hierarchies (dimensions and coding scheme of informality processes as set out in Table 1). These saturated in seven hierarchies: economic (Eco), legal (Leg), social (Soc), political (Pol), cultural (Cul), ethical (Eth) and labor (Lab) ( Table 2). This categorization brings us closer to the empirical level, that is, to be able to analyze as concrete something originally defined as purely theoretical. This procedure can readily be applied to other lines of research and objects of study such as corruption, globalization, social economy or job insecurity.

**Table 2.** Range and relative weighting of each category in Google Scholar.

*j*	Dimensions	*D _j_*	% * PD _j_*
**1**	Economic	3,283	87
**2**	Legal	2,669	71
**3**	Social	2,110	56
**4**	Political	2,006	53
**5**	Cultural	89	2
**6**	Ethical	1,969	52
**7**	Labor	1,336	35

**Source: compiled by the author. J is the number of counts in each dimension; *PD _j_* is the relative weight of each dimension j, expressed as a %; Dj is the range of each dimension j.**

The frequency count ( *f _i_*) was carried out in Google Scholar. The absolute frequencies, *f _i_*, in Table 1 are expressed according to *f _kj_*, which refers to the number of counts of a dimension, ***j*** (j = 1, …, 7), within each bibliographic citation, ***k***. The extended sum of all ***z*** (the sum from 1 to 102) elements that contain dimension ***j*** gives the range of each dimension ( Equation 1); by dividing by the total frequencies, their relative weighting is derived ( Equation 2). The values obtained from these formulas are listed in Table 2. 


$$D_{j}=\sum_{k=1}^{z}f_{kj}\;[\text{Equation}\;1]$$

Where,

*D _j_* is the range of each dimension ***j***.

*f _kj_* is the frequency of the citation ***k*** of a single dimension ***j.***


$$\%\;PD_{j}=\frac{D_{j}}{\sum_{i=1}^{x}f_{i}}100\;[\text{Equation}\;2]$$

Where,

*PD _j_* is the relative weight of each dimension ***j***, expressed as a %

*f _i_* is the absolute frequency of the Google count of every ***k*** concept.

## Results

Table 8 ( Luque, 2021) provides the broad spectrum of published definitions of informal economy appearing in the literature (compiled between 12/09/2020 and 24/11/2020), together with their authors and the number of times they have been cited in the literature to date.

Table 2 more clearly exemplifies the data distribution. The number of hierarchical dimensions and the relative weight of each association in the Google Scholar frequency count is detailed below.

As can be seen, the economic dimension has the greatest range in Google Scholar, with 3,276 units of frequency; the social, political, and ethical hierarchies are of similar relative importance at approximately 50%, while the cultural construct is of residual importance according to this analysis.

### Governing dominions and most significant connections: a tetrad model

The importance of the above parameters lies in their ability to support the dimensions that comprise the processes of informal economy. To this end, it is necessary to list a higher order in the distribution of UAs based on the number of domains associated with each of the 102 concepts. 

The information derived from Table 3, shows that the ends of the curve correspond to the theoretical constructs of least relative importance (around 1% of the count in Google Scholar). These have, on the one hand, the largest number of possible dimensions, and on the other, are confined to a single dimension. In contrast, the greatest weighting corresponds to the UAs found within any four hierarchies —tetrads— which group together 26 of these UAs of higher-order ( Table 3). These are highlighted in gray in Table 8, which also includes all of the analyzed concepts. Tetrads have 12,1 % more weighting (and therefore more citations) than triads in the Google Scholar count, despite containing 10 UAs fewer. The fact that the most likely association of UAs is made up of quartets, and not of dyads or triads, implies the need for a more sophisticated study of category interaction. This is set out below.

**Table 3.** Distribution of the units of analysis (UAs) and percentage weighting.

No. of dimensions	No. of UAs	% Weighting in the count	Distribution
**7**	2	1,5	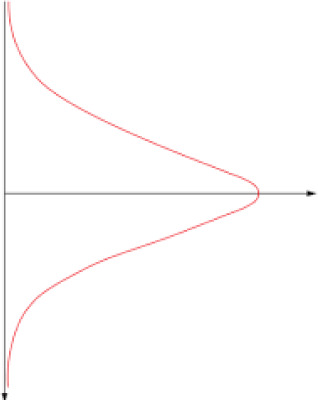
**6**	1	0,3	
**5**	11	10,5	
**4**	26	42,8	
**3**	36	30,7	
**2**	23	13,4	
**1**	3	0,8	
	Total = 102	Total = 100 %	

Source: compiled by the author.

The tetrad model involves the combination of seven categories, in groups of four, resulting in 35 possible combinations. However, from the first-order categorization ( Table 3), 10 of these emerge as the most likely, which are represented in Table 4. The parameters associated with each individual category correspond to their relative weighting, expressed as a percentage. Processes of informal economy are mainly represented by the Eco-Leg-Pol-Eth and Eco-Leg-Soc-Lab tetrads. Social status has a diminished presence here and the ethical and labor dimensions are weakly defined, if at all (for example, tetrads 10, 16 and 33 with weightings of 3.7, 1.4 and 0.4 respectively); cultural status is not involved in these processes.

**Table 4.** Tetrads model.

Tetrads		Number of UAs included	Weighting fraction (%)
**1**	Economic-Legal-Social-Political	4	9,9
**3**	Economic-Legal-Social-Ethical	3	3,5
**4**	Economic-Legal-Social-Labor	5	17,2
**6**	Economic-Legal-Political-Ethical	4	39,6
**7**	Economic-Legal-Political-Labor	2	8,3
**10**	Economic-Legal-Ethical-Labor	1	3,7
**12**	Economic-Social-Political-Ethical	2	7,5
**16**	Economic-Social-Ethical-Labor	1	1,4
**26**	Legal-Social-Ethical-Labor	3	8,5
**33**	Social-Political-Ethical-Labor	1	0,4
		**Total = 26**	**Total = 100%**

Source: compiled by the author

The associations mentioned above are the extremes of a continuum, from which it is inferred that the strongest connection is established between the economic and legal dimensions, while the weakest interaction is the between labor and ethical. On the other hand, the political dimension contains the fewest interactions with other categories.

In the next step, taking into account the sum total of each column, the domains that govern the processes of the informal economy are the economic, legal, and ethical, whereas the political, social, and labor domains do not control the evolution of this economy, or do so only residually. In accordance with ILO Recommendation No. 204 on the transition from the informal to the formal economy ^2^ ( ILO, 2015), the creation and sustainability of decent enterprises and jobs should be facilitated and promoted, as well as the prevention of the informalization of jobs.

If we apply combinatorial analysis, the grouping of seven elements by fours provides 35 possible tetrads, such as those shown in Table 5. Of these, only 10 have been shown in the study (“appearing”) and excluding the remaining 25 (“not appearing”). This information was collected by taking the individual contribution of each dimension or % *PD _j_* and taking the algebraic sum of the tetrad to obtain its relative importance in decreasing order.

**Table 5.** Model of tetrads across the seven categories with total (%).

Tetrads		Economic (Eco)	Legal (Leg)	Social (Soc)	Political (Pol)	Cultural (Cul)	Ethical (Eth)	Labor (Lab)	Relative importance (⁰/1)
**Appearing**	1	0,87	0,71	0,56	0,53				2,67
	3	0,87	0,71	0,56			0,52		2,66
	6	0,87	0,71		0,53		0,52		2,63
	4	0,87	0,71	0,56				0,35	2,49
	12	0,87		0,56	0,53		0,52		2,48
	7	0,87	0,71		0,53			0,35	2,46
	10	0,87	0,71				0,52	0,35	2,45
	16	0,87		0,56			0,52	0,35	2,30
	26		0,71	0,56			0,52	0,35	2,14
	33			0,56	0,53		0,52	0,35	1,96
**Not appearing**	2	0,87	0,71	0,56		0,02			2,16
	22		0,71	0,56	0,53		0,52		2,32
	13	0,87		0,56	0,53			0,35	2,31
	19	0,87			0,53		0,52	0,35	2,27
	23		0,71	0,56	0,53			0,35	2,15
	5	0,87	0,71		0,53	0,02			2,13
	8	0,87	0,71			0,02	0,52		2,12
	29		0,71		0,53		0,52	0,35	2,11
	11	0,87		0,56	0,53	0,02			1,98
	14	0,87		0,56		0,02	0,52		1,97
	9	0,87	0,71			0,02		0,35	1,95
	17	0,87			0,53	0,02	0,52		1,94
	21		0,71	0,56	0,53	0,02			1,82
	24		0,71	0,56		0,02	0,52		1,81
	15	0,87		0,56		0,02		0,35	1,80
	27		0,71		0,53	0,02	0,52		1,78
	18	0,87			0,53	0,02		0,35	1,77
	20	0,87				0,02	0,52	0,35	1,76
	25		0,71	0,56		0,02		0,35	1,64
	31			0,56	0,53	0,02	0,52		1,63
	28		0,71		0,53	0,02		0,35	1,61
	30		0,71			0,02	0,52	0,35	1,60
	32			0,56	0,53	0,02		0,35	1,46
	34			0,56		0,02	0,52	0,35	1,45
	35				0,53	0,02	0,52	0,35	1,42

Source: compiled by the author

Table 6 shows that the political and labor categories vectorize the processes of informality only residually, evidencing the reality of these processes in which labor, owing to its direct relationship to informality, and the political architecture, which is key to thier conceptualization and solution, have less importance than might be expected. What is more, the cultural dimension is of least significance, which also precludes one of the principal solutions to the problem, that is, the knowledge of the traditions in each community and state. With such traditions come aspects such as religion, corruption, coups d'état, endogenous political and social instability, identity, etc. It should be noted that the acceptance of employment and its conditions, including those of an informal nature, are directly related to the expression of human activities and thoughts, which gives intangible significance to a reality that is not always quantifiable.

**Table 6.** Mirror values.

	Economic	Legal	Social	Political	Cultural	Ethical	Labor
Appearing	6,96	4,97	3,92	2,65	0,00	3,64	2,10
Not appearing	10,44	9,23	7,28	7,95	0,40	6,76	4,90

Source: compiled by the author

This fact leads us to two complementary positions: on the one hand, to the induction of the current concept of informality as that which results from the most commonly used approaches within the four-fold economic-legal-political-ethical dimension; and to complement this with the definition of greater weighting ( Table 7).

**Table 7.** Definitions with greater weight.

Becker, 2004a, p. 10	Informal work arrangements are a rational response by micro-entrepreneurs to over-regulation by government bureaucracies.	386	Economic Legal Political Ethical
Henry, 1978	An offshoot of capitalism	101	Economic Legal Political Ethical

Source: compiled by the author

**Table 8.** Definitions of informal economy (tetrads in grey color).

Author	Concepts from the authors	Freq.	Dimensions
Polanyi, 1957	Individual networks for the exchange of services and products in scarcity that follow the principle of reciprocity, client structures within state bureaucracy (redistribution) and illicit production of goods and services distributed through the black market.	87	Economic Social Political Ethical
Klein & Tokman, 2000, pp. 7–30	Activities not recognized, registered, protected or regulated by public authorities.	25	Economic Legal Political
Hart, 1997, pp. 61–89	Marginal activities—other than the formal and non-formal sector—that provide income to the poor and a safety-net in times of crisis.	35	Economic Social Political Ethical
Moser, 1978, pp. 1041–1064	Economic units (micro-businesses) and subordinate workers that help to reduce input and labor costs, thereby increasing the competitiveness of big business.	98	Economic Legal Political Labor
Vivancos, 1989	Heterogeneous group of production and marketing activities not subject to accountancy controls or that may be openly illegal.	83	Economic Legal Ethical
Feige, 1990, pp. 989–1002	Economic activities that ignore the costs of complying with laws and administrative regulations governing "ownership relationships, commercial licensing, employment contracts, liabilities, financial credit and social security systems" and are excluded from all such protections.	97	Economic Legal Labor
Portes, 1995	The forms of subordination of labor to capital and an expression of the modes of production carried out at the level of the urban economy, similar to that previously performed in the area of the agricultural economy.	171	Economic Legal Social Labor
Castells, 1998	Systematic effort by companies in the formal sector to evade or disassociate themselves from the legal requirements of labor protection, through the method of outsourcing that lacks formal employment benefits, in order to substantially reduce labor costs and tackle cut-throat global competition.	51	Economic Legal Labor
de Soto, 2000	It is made up of "intrepid" micro-entrepreneurs who choose to work informally to avoid the costs, time and effort of formal registration, and also of those who would need property rights to make their assets legally recognized.	39	Economic Legal
Gomez, 2007, pp. 7–30	Ability to absorb self-created employment and its relationship with the modern production sector, which is the result of changes in the economy and the labor market.	8	Economic Legal Political Medioambiental Cultural Ethical Labor
Parra, 2003	A set of economic activities carried out by workers and economic units which, both in law and in practice, are insufficiently covered by formal systems. The activities of these individuals and companies are not covered by legislation, which means that they operate outside the law; or they are not covered in practice, i.e. that legal regulations are not applied or not enforced; or the legislation itself causes incompliance because it is seen as inadequate, cumbersome or it imposes excessive costs.	37	Economic Legal Political Labor
Méndez, 2002	Individual and commercial subsistence activities, dislocated from their social base.	2	Economic Social
Icaza & Tiribia, 2003	Activities that, although carried out within social sectors, do not fall within the classification of social economy, but rather within the informal economy.	7	Social
WIEGO, 2003	All of the economic units, activities and workers thus defined, as well as the results of their production, that together form, both domestically and globally, the broad base of the workforce and the economy.	68	Economic Social Labor
International Labour Organization, 2010	All of the economic activities carried out by workers and economic units which—for both legal and practical purposes—are not covered or are insufficiently covered by formal systems.	9	Economic Legal Social Political Labor
Bertranou, 2007	Self-employed workers performing subsistence activities alongside others who carry out unsalaried activities that generate income and a certain contributory capacity.	2	Economic Labor
Iranzo, 2007	Employment activities that are not legally registered and therefore do not pay taxes and have little chance of economic growth.	7	Economic Legal Labor
Programa Regional del Empleo para América Latina y el Caribe, 2007	An ensemble of small-scale economic activities engaged in the production and distribution of goods and services with a weak capital-to-work ratio and low level of profitability and technology, and that are characterized by precariousness and vulnerability to the prevailing economic conditions in each country.	6	Economic Legal Social Labor
Moyano *et al*., 2008, pp. 693–701	A growing and diverse group of workers and enterprises (urban or rural) operating in non-regulated areas.	7	Legal Labor
Chen, 2012	Principal source of jobs and livelihoods in most cities of the developing world.	11	Economic Social Political
Haller, 2014	Unregistered or lawful economic activities; the inability of the public administration to enforce the corresponding regulations across companies and the whole population.	4	Economic Legal Social Ethical
Quijano, 2004, pp. 55–83	Those economic activities that are not recorded in the official statistics and that operate outside of administrative controls.	2	Economic Legal
( ILO, 2003, p.10)	All activities that, in law or in practice, do not fall within the remit of formal mechanisms.	8	Economic Legal
Huggins, 2007	Self-employed activities, carried out in small, unregistered businesses.	21	Economic Legal
Dominguez, 2004, pp. 10–32	Group of activities that, for a variety of reasons, remain outside standard information systems, such as national accounting and auditing, tax records, employment statistics, legal records or the economic conceptual framework itself.	3	Economic Legal
Boyd, 2017	A wide range of activities, from work mainly concerned with subsistence to sophisticated tasks that require high-level technical skills.	3	Social
Cervantes *et al.*, 2008	Entrepreneurial or individual activity not subject to legislation or lying outside of the law—at least for practical purposes—also those to which the law is not applied, or incompliance is common; especially where legislation is deficient, excessively onerous or costly.	32	Economic Legal
Moccerro, 2001	Contraction of economic activity and non-payment of employer contributions (and other forms of evasion of responsibilities) that offer a source of finance to companies and artificially raise profitability.	47	Economic Legal Social Ethical Labor
Gallart, 2005, pp. 36–47	An income-generating process that is not regulated by social institutions in a legal environment in which similar activities are usually regulated.	6	Economic Legal Social Political
Converti, 2017	Economic activity that is not taxed or regulated by a government, which contrasts with the formal economy including legitimate economic activity under national law.	9	Economic Legal Political
Chen, 2005, pp. 18–27	It consists of all forms of "informal work", i.e., without employment or social protection, both within and outside of informal businesses, including self- employment in small, unregistered businesses and wage labor in unprotected jobs.	18	Economic Legal Social Labor
Breman, 1996	Relationships between employers and workers are governed by a wide range of social customs and traditions that mitigate the sense of insecurity of poorer workers.	96	Legal Social Ethical Labor
Henriques & Herr, 2007, p.43	Is where large numbers of poor people work. In addition to low incomes, informal work is most often characterized by poor working conditions, a lack of formal employment contracts, and very limited or no social protection. Particularly women are affected, since they form the majority of workers in this part of the economy.	33	Economic Legal Political Ethical Labor
Tripp, 1997, p. 2	Economic activities that are subject to regulation, but which, in fact, operate outside of government controls.	8	Economic Legal Political Ethical Labor
Sassen, 1993	Those revenue-generating activities that are carried out beyond the state's regulatory framework and are analogous to others within that framework.	59	Economic Legal
Gershuny, 1979, pp. 3–15	It contains a diverse collection of activities that can be divided into three ambits: home, community, and underground. The latter covers a wide range, from outright theft to tax evasion.	7	Social Political Ethical Labor
Mukhija & Loukaitou-Sideris, 2015	Occur in defiance of rules, ordinances, and regulations, out of necessity, or because of opportunity.	15	Legal Social Political
Castells & Portes, 1989b, pp. 11–37	The Non-formal and unregulated part of the market economy that produces goods and services for sale or for other forms of remuneration.	62	Economic Legal Social
Amin & Singh, 2002	Self-employed workers assisted by family members or family workers.	32	Social Labor
Chandra & Pratap, 2001	Unincorporated enterprises and cottage industries (other than organized ones) that are not regulated by law and do not present annual accounts or balance sheets constitute the unorganized sector.	54	Economic Legal Social Political
Geertz, 1963	It is both an economic institution and a way of life, a general mode of commercial activity that encompasses all aspects of society.	5	Economic Social Political
Fernandez & Scheffner, 2006	These are transactions in which the state does not provide protection or receive a "cut"; therefore, the relationship between the informal economy and the state is, by definition, inevitably one of conflict.	23	Economic Legal Social Political
Apressyan, 1997, pp. 1561–1570	Illegal use of state, financial or material resources, or outright theft, essentially parasitic in nature.	75	Economic Legal Political Ethical
Chakraborty, 1997, pp. 1529–1538	It denotes the process of the clandestine selling of scarce or rationed commodities at inflated prices.	68	Economic Social Ethical
Daza, 2015, pp. 5–17	Economic activities of workers and economic units that—for legal or practical purposes—are not covered or insufficiently covered by formal agreements	29	Economic Legal Labor
Luna, 2006, pp. 57–63	It consists of economic activities with different levels of complexity, with workers that are mainly self-employed and trade in basic products such as food or provide basic transport services.	17	Economic Social Labor
Sheinbaum & Lomnitz, 2005	Those aspects that allow the poor to subsist and the middle and upper classes to maintain their social status and privileges	23	Social Political Ethical Labor
Levenson & Maloney, 1998, pp. 11–21	It is the result of producers and traders who choose to operate informally after weighing the costs and benefits of informality versus formality, especially opportunistic informal producers and traders.	56	Economic Social
Watson, 2009, pp. 151–168	Anti-poverty policies and regulations and government systems that exclude the poorest informal producers and traders from access to formal employment, basic urban services and space in the city to live and work.	17	Social Political Labor
Centre Emile Berheim CEB, 2009, pp. 15–36	It helps to organize society.	32	Social Political
Cortés, 2000, pp. 592–618	Economic activities with low levels of productivity that employ family members; low-paying jobs; occasional employment; urban poverty; and belts of misery on the margins of major Latin American cities.	47	Economic Legal Social Political Cultural Ethical Labor
Junguito & Caballero, 1978	Unregistered (or over-registered) productive activities featuring in the national accounts in the agriculture, industry, trade, mining and construction sectors. These originated in the production of small-scale goods and services such as those of the agricultural mini-trader, the street trader, etc. and are carried out outside the law.	87	Economic Legal Social Political Ethical
Williams & Youssef, 2014, pp. 41–53	Activities in which monetary transactions are not declared to the state for taxation, social security or labor law purposes, but which are legal in all other respects.	15	Economic Legal Social Ethical
Charmes, 1992, pp. 316–408	Lawful but unregistered economic activities; although non-registration is not always synonymous with illegality, but rather may reflect the inability of the public administration to enforce the corresponding regulations for the whole population.	65	Economic Legal Social Political Ethical
Neffa, 2009, pp. 15–18	It also includes all workers without social protection (generally referred to as unregistered) regardless of whether they are in small, medium or large enterprises, or whether urban or rural.	32	Economic Legal Social Political Labor
UN women, 2020	Circumstances that lack the protections afforded by employment and social benefit laws such as pensions, health insurance or paid sickness allowances.	65	Economic Legal Social Labor
Coraggio & San Pablo, 1991	Economic activities (in the sense of producing goods and services or using up scarce resources) carried out by individual or collectives that continually rely on their own labor.	76	Economic Social
de Sousa, 2004, pp. 141–192	Activities not regulated by the State	37	Legal Political
Quijano, 1998, p. 92	A decisive factor is group identity (ethnic, regional, religious, family and political) as the main cohesive element in economic activity.	73	Economic Social Political
Cacciamali, 2000, pp. 95–110	Workers who carry out activities characterized by instability and low levels of remuneration.	34	Economic Social Labor
Bastidas & Richer, 2001, pp. 13–20	It is the least productive employment area, with a workforce that failed to enter the modern labor market. The marginalized informal sector was destined to be re- absorbed under the pressure of global economic growth and public policy.	6	Economic Social Political
Waisgrais, 2001, pp. 15–32	It is part of a segmentation process in the labor market, including self-employed activities, domestic service or even unprotected employees.	12	Economic Ethical Labor
Carida & Lacabana, 1989, pp. 25–48	Small-scale dependent activities, carried out with or without paid workers, which are characterized by low-level organization and technology and whose fundamental objective is to create jobs and generate income for their participants.	23	Economic Social Ethical Labor
Albalate & Bacardit, 2014, pp. 20–56	Those activities that, being commercial, are not subject to labor and legal controls.	7	Economic Legal Social
Escobar, 2014, pp. 16–67	Activities that require greater resources and a longer-term outlook are subject to all kinds of uncertainties and risks.	3	Economic Social
CIPPEC, 2009, pp. 29–96	They are low-quality employment activities that involve irregular or low incomes, long working hours and lack of access to information, markets, finance, training and technology.	12	Economic Social Political Cultural Ethical Labor
Díaz & Gálvez, 2003, pp. 18–36	Heterogeneous set of means of production and employment, interrelated with each other, but with different dynamics and influenced both by general policies and by policies specific to some of them.	6	Economic Political Labor
Saavedra, 2007, pp. 207–341	It is the ratio of self-employment activities to full employment, and of workers without pension or social security contribution to all workers.	19	Economic Legal Social Labor
Jaller, 2007, pp. 13–54	That which includes small producers, processing companies, traders and service providers, as well as legal and illegal activities involving a large number of artisans.	23	Economic Social
Arqué, 2005, pp. 5–41	It is an income-generating process characterized by its avoidance of the regulations of social institutions.	29	Economic Legal Social
Rodríguez, 2012, pp. 48–157	Inability of economies to create a sufficient number of good quality jobs to absorb the workforce.	15	Economic Ethical Labor
Azaïs *et al*., 2012, pp. 81–163	They are street activities.	32	Economic Social Political
Webb *et al*., 2013, pág. 598	The informal economy consists of economic activities that occur beyond the boundaries of formal institutions but remain within informal institutional boundaries for large segments of society.	77	Economic Legal Social Political
de Soto, 1989, p. 11	They are not “antisocial in intent”	90	Social Political Ethical
Webb *et al*., 2009	From a policy point of view, another potentially effective approach to limiting the scope of the informal economy is to pressure big business in the formal economy to break its links with suppliers that operate illicitly.	12	Economic Social Political
Rutherford & Buller, 2007	Activities, which, while illegal, remain legitimate for large portions of society.	34	Social Ethical
Centeno & Portes, 2006	The production of legitimate goods using processes that are not fully legal.	22	Economic Cultural Ethical
Nichter & Goldmark, 2009	Economic activities that are unregistered yet produce legal products.	29	Economic Legal Ethical
LaPorta & Schleifer, 2008, p. 1	The informal economy is defined as “economic activity that [is] conducted by unregistered firms or by registered firms that are hidden from taxation.”	49	Economic Legal Ethical
Venkatesh, 2006	Informal, underground, or “off-the-books” economic activity as characterized by either illicit practices (products or processes) or illicit exchanges.	41	Economic Ethical
Becker, 2004a, p. 10	Informal work arrangements are a rational response by micro-entrepreneurs to over-regulation by government bureaucracies.	386	Economic Legal Political Ethical
Andrews *et al*., 2011, p. 7	Economic activities and transactions that are sufficiently hidden so that they are unmeasured or untaxed, and it is presumed that economic agents are at least passively aware that bringing these activities to the attention of authorities would imply tax or other legal consequences.	50	Economic Political Ethical
Schneider & Buehn, 2009, p. 2	All market-based legal production of goods and services that are deliberately concealed from public authorities for the following reasons: (1) to avoid payment of income, value added or other taxes, (2) to avoid payment of social security contributions, (3) to avoid having to meet certain legal labor market standards, such as minimum wages, maximum working hours, safety standards, etc., and (4) to avoid complying with certain administrative procedures, such as completing statistical questionnaires or other administrative forms.	37	Economic Legal Social Ethical
Cortellese, 2015	What should be formal according to the law of a state but is not. We will limit the analysis to the activities that affect public revenue and expenditure and, at the same time, cause a distortion in the market.	21	Legal Political
Randhawa, 2017	The informal sector is characterized by easy entry and exit, small scale of operation, few employees and inability to access services in the formal sector, including finance, power and water supply. This keeps down the level of capital invested. As a result, informal enterprises generally use older cheaper technologies in the production of goods and services.	18	Economic Social Political Ethical Labor
Carrasco, 2004, pp. 128–163	It has as one of its characteristics the "lack of an employment relationship", this does not necessarily mean that the worker does not have a "boss".	20	Labor
Brown & McGranahan, 2016, pp. 91–142	It includes economic activities that fall largely outside the scope of official regulation, either because the regulations do not apply or through a combination of their weak application and evasion.	9	Economic Legal Political
International Labour Organization, 2002b, pp. 11–37	Informal activities that are not subject to accountancy, such as production for personal consumption, paid domestic activities in private households and door-to- door work.	5	Economic Ethical
Chen, 2017, pp. 3–15	A large proportion of economic units and workers remain outside the world of regulated economic activities and protected employment relationships.	6	Economic Legal
Becker, 2004b, pp. 150–184	It is largely characterized by low-income requirements in terms of capital and professional qualifications; a small scale of operations; skills often acquired outside of formal education; labor-intensive production methods and adapted technology.	14	Economic Social Ethical
Losby *et al*., 2002, p. 1	The informal economy refers to a series of activities that, by occurring outside the arena of the normal, regulated economy, escape official record keeping.	22	Economic Legal Ethical
Levitan & Feldman, 1991	Economy that includes monetary and non-monetary aspects.	23	Economic Social Political
Gerber, 1999, p. 1	The informal economy "has served as a crucial survival strategy for the poor, as a significant provider of jobs to the unemployed, as a training ground for entrepreneurs, a source of new businesses, and as part of a cost reducing strategy for modern businesses."	49	Social Political Labor
Williams & Nadin, 2010, p. 363	Paid production, as well as the sale of goods and services that are legitimate in all respects, despite not being registered or hidden from the state for tax and/or profit purposes.	19	Economic Legal Social Political Ethical
International Labour Organization, 2002a	All economic activities by workers and economic units that are—in law or in practice—not covered or insufficiently covered by formal arrangements’	19	Legal Social Ethical Labor
Smith, 1994, p. 18	Market-based production of goods and services, whether legal or illegal, that escapes detection in the official estimates of GDP.	64	Economic Legal Social Political Ethical
Schneider, 2014, p. 228	All market-based legal production of goods and services that are deliberately concealed from public authorities.	13	Economic Legal Ethical
Bruton *et al*., 2012, p. 1	It is the final frontier of the management domain.	3	Economic Ethical
Schneider, 2005	Expected to influence the tax system and its structure, the efficiency of resource allocation between sectors, and the official economy in a dynamic sense. People engage in shadow economic activity for a variety of reasons, among the most important of which are government actions, most notably taxation and regulation.	60	Economic Legal Ethical Labor
Igudia *et al*., 2016, p. 3	Consisting of three elements: informal employment (i.e., those doing informal types of job, regardless of the location of enterprise or operation); employment in the informal economy (i.e., those working in informal sector enterprises, regardless of the type of job done); and all legal activities that contribute to GDP but which do not figure in official statistics, for various reasons.	17	Economic Legal Social Political Labor
Henry, 1978	An offshoot of capitalism	101	Economic Legal Political Ethical
Williams, 2013, p. 263	A result of the structured development within the capitalist mode of production, the ‘deregulated global economy’, and under-regulation economies.	79	Economic Legal Political Ethical

Source: compiled by the author.

## Conclusions

The processes of informality present real problems, but not in equal measure for all stakeholders. All societies experience them and suffer their consequences to a greater or lesser extent, although it should be remembered that the vast majority of people are immersed in them not out of choice, but rather as a means of subsistence. Criminalizing informality without providing assistance or solutions only highlights the failings of a current globalized society beset with vested interests and lacking the means of redress or misusing the available resources in developmentalist projects such as strengthening the army (together with the constant drain of buying materiel), roadbuilding, and tax exemptions for polluting vehicles. This is compounded by the establishment and defense of policies that move away from the processes of social economy in favor of protectionist legislation that force many out of the system and necessitate their involvement in the informal economy. The promulgation of the sustainable development goals is a first step in establishing decent work as the standard and not the exception. The political and social commitment they represent should be striven for, so that this battery of measures might not be corrupted by the surge of vested interests, as has happened with the processes of corporate social responsibility (full of good intentions without any enforceability beyond its positive impact on corporate dividends or the public image of management).

The categorization of informality was saturated in the seven basic areas of economic, legal, social, political, cultural, ethical, and labor dimensions. From the amalgamation of these, the meta-concept of the informal economy—not yet described in the literature—can be derived. However, not all categories contribute equally. The results show that a tetrad model is best suited to describe the sophisticated connections that exist between the different categories. Of these, the cultural dimension has been the most overlooked in academic publications, confirming its devaluation in productivist views, despite the myriad opportunities it offers; meanwhile, the legal aspect is most strongly rooted in published conceptualizations (excepting the economic dimension, which dominates for obvious reasons) in trying to eliminate informality through the exercise of the rule of law and regulatory frameworks, including the occasional promotion of institutional violence. These results are consistent with both the number of connections that are established between these categories and their individual inputs. Perhaps most surprising is the relative lack of importance attached to the social and labor dimensions, which highlights the contradiction of an academic community that represents the civilized world yet appears to be closer to the abyss than to the solution.

## Data availability

### Underlying data

Figshare: Analysis of the concept of informal economy through 102 definitions: legality or necessity. https://doi.org/10.5281/zenodo.5525159. ( Luque, 2021).

The project contains the following underlying data:

- Table 1 and Table 8 Search chain and Definitions of informal economy Arturo Luque.pdf. (a large group of 102 linked units of analysis (UA) was obtained, which was used to define the concept under study description of data).

Data are available under the terms of the Creative Commons Attribution 4.0 International license (CC-BY 4.0).

## Footnotes

^1 ^27 Spanish multinationals pay a corporation tax of 0.3% of their profit worldwide, and the 134 large multinationals, with their headquarters in Spain, presented a profit of 91.894 billion USD in 2016 and paid 11.594 billion USD tax worldwide (12.6%). In addition, 36.6% of Spanish multinationals (49) obtain a net profit of 31.4%, yet contribute only 6.4% of global tax—the average effective rate being 3.1%—according to agency tax data, 2019. Retrived from Agencia Tributaria (2019)

^2 ^Different ILO instruments have been proposed, such as the Fundamental Conventions on 1) Forced Labour, 1930 (No. 29), and 2014 Protocol, 2) The Freedom of Association and Protection of the Right to Organise, 1948 (No. 87), 3) The Right to Organise and Collective Bargaining Convention (4) Equal Remuneration, 1951 (No. 100), 5) The Abolition of Forced Labour, 1957 (No. 105), 6) Discrimination (Employment and Occupation), 1958 (No. 111), 7) Minimum Age, 1973 (No. 138), 8) Worst Forms of Child Labour, 1999 (No. 182); and the Governance Conventions on 1) Labour Inspection, 1947 (No. 81), 2) Employment Policy, 1964 (No. 122), 3) Labour Inspection (Agriculture), 1969 (No. 129), 4) Tripartite Consultation (International Labour Standards), 1976 (No. 144). Similarly, there are the UN instruments, of which the most relevant are 1) The Universal Declaration of Human Rights, 1948, 2) The International Covenant on Economic, Social and Cultural Rights, 1966, 3) The International Covenant on Civil and Political Rights, 1966, and 4) The International Convention on the Protection of the Rights of All Migrant Workers and Members of Their Families, 1990
