# Supplementary material for: Analysis of the concept of informal economy through 102 definitions: legality or necessity
Source: Open Res Eur. 2022 Jan 18;1:134. Originally published 2021 Nov 4. [Version 2] doi: 10.12688/openreseurope.13990.2 (PMC10521053; doi:10.12688/openreseurope.13990.2)
Supplement: Peer review history

# Reviewer report: Reviewer response for version 2

**DOI:** 10.21956/openreseurope.15513.r28393

**Published:** 2022-02-01

**Authors:** Edisson Coba

**Affiliations:** 1 Universidad Técnica de Ambato, Ambato, Ecuador

I congratulate the authors who have accepted the suggestions made and the article is much better.

If applicable, is the statistical analysis and its interpretation appropriate?

Yes

Is the study design appropriate and is the work technically sound?

Yes

Is the work clearly and accurately presented and does it engage with the current literature?

Yes

Are the conclusions drawn adequately supported by the results?

Yes

Are sufficient details of methods and analysis provided to allow replication by others?

Yes

Are all the source data and materials underlying the results available?

Yes

Reviewer Expertise:

Social responsability; Popular and Solidary Economy; Accounting and Finance

I confirm that I have read this submission and believe that I have an appropriate level of expertise to confirm that it is of an acceptable scientific standard.

# Reviewer report: Reviewer response for version 2

**DOI:** 10.21956/openreseurope.15513.r28394

**Published:** 2022-01-19

**Authors:** Fahri Fahri Özsungur

**Affiliations:** 1 Department of Labor Economics and Industrial Relations, Mersin University, Mersin, Turkey

All necessary revisions were made. I congratulate the authors.

If applicable, is the statistical analysis and its interpretation appropriate?

Yes

Is the study design appropriate and is the work technically sound?

Yes

Is the work clearly and accurately presented and does it engage with the current literature?

Yes

Are the conclusions drawn adequately supported by the results?

Yes

Are sufficient details of methods and analysis provided to allow replication by others?

Yes

Are all the source data and materials underlying the results available?

Yes

Reviewer Expertise:

Business, law, finance, social work, social policy, women studies

I confirm that I have read this submission and believe that I have an appropriate level of expertise to confirm that it is of an acceptable scientific standard.

# Reviewer report: Reviewer response for version 1

**DOI:** 10.21956/openreseurope.15076.r27989

**Published:** 2021-11-18

**Authors:** Edisson Coba

**Affiliations:** 1 Universidad Técnica de Ambato, Ambato, Ecuador

The article shows in an interesting way the different meanings of informality, and the little agreement that exists in the theory and its practical implications.

 One of the observations is that in the Economic dimension the search chain: “weakening of control of regulatory bodies” would seem to deal with more legal aspects as they are control bodies. Similarly, the search string 'promotion and conservation of ancestral traditions' should be treated as a cultural rather than a political aspect.

 It should be clarified in the methodology section that the search engine used was Google Scholar from the beginning. The section on Sequencing the parameters of the informal economy, should be mentioned earlier and not later. This could avoid confusion when understanding how to select articles in indexed Latindex and Web of Science journals.

 The explanations in tables 3, 4 and 5 should have a greater depth of analysis regarding the impact of the results obtained; and not only highlight the frequencies and percentages of tabulation. It would be of great value to know the author's position regarding these results and to have some discussions about it.

 In Table 8 about of Definitions of the informal economy, it is very subjective to differentiate a definition that has an ethical, cultural and political dimension. Despite the description of the definitions of each dimension, it is advisable to be more precise to specify how to interpret that a definition is framed in these three dimensions.

If applicable, is the statistical analysis and its interpretation appropriate?

Yes

Is the study design appropriate and is the work technically sound?

Yes

Is the work clearly and accurately presented and does it engage with the current literature?

Yes

Are the conclusions drawn adequately supported by the results?

Yes

Are sufficient details of methods and analysis provided to allow replication by others?

Yes

Are all the source data and materials underlying the results available?

Yes

Reviewer Expertise:

Social responsability; Popular and Solidary Economy; Accounting and Finance

I confirm that I have read this submission and believe that I have an appropriate level of expertise to confirm that it is of an acceptable scientific standard, however I have significant reservations, as outlined above.

# Response

**Published:** 2021-12-08

**Authors:** Arturo Luque

**Affiliations:** Universidad Técnica de Manabí, Ecuador

Dear Mr. Edisson Coba (https://open-research-europe.ec.europa.eu/articles/1-134)

 First of all, I wish to thank you for the thoroughness, kindness and courtesy you have shown in your review.  I fully understand and value the work of a reviewer, which in many cases receives no other reward than the professional satisfaction of carrying out a vital task and the respect of fellow academics who, after months (or years) of work on a project, submit their papers for evaluation. As the lead author of this manuscript, I too review a number of articles throughout the year. In this case, your indications and advice seem entirely constructive, with the clear purpose of improving the study. Understandably, we have had to balance your comments with some of the recommendations of the other three reviewers in order to avoid contradictions in the text. I enjoy the challenge of incorporating corrections and suggestions and the process, of course, helps all writers to grow, learn and avoid future mistakes. Even where entire passages have needed to be altered in accordance with your review, I have diligently attempted to respond to your recommendations as enumerated below:

 The following observations have been taken into consideration and duly corrected: *“One of the observations is that in the Economic dimension the search chain: “weakening of control of regulatory bodies” would seem to deal with more legal aspects as they are control bodies. Similarly, the search string 'promotion and conservation of ancestral traditions' should be treated as a cultural rather than a political aspect.”*

 According to reviewer 2, *“It should be clarified in the methodology section that the search engine used was Google Scholar from the beginning”*, but this point is already clarified in the methodology: “A search was carried out for definitions of the informal economy and related constructs through the existing literature using indexed databases, which in turn were part of Google Scholar ( https://scholar.google.com/)”, and “The selection of the indexed academic databases to be used were: Web of Science, Scopus and Latindex with no time limit on date of publication or type of paper. Both English language and Spanish publications were included in the search between 12/09/2020 and 24/11/2020. 102 definitions were found by this method between 12/09/2020 and 24/11/2020”.

 The definitions were searched in three databases (Web of Science, Scopus and Latindex) and once they were found it was verified that they were also indexed in Google Scholar.  Consequently, we saw no need to provide further clarification.  

 For the compilation of Table Nº8, the dimensions and methodology were previously established and justified. This was done by drawing on high-impact articles that validate the method as well as finding justifications in the existing specific literature. Definitions as such are not interpreted but rather categorized in relation to dimensions. There is no doubt that, as the study points out, only an approximation is possible to many of the current trends in informal economy processes. These are not unambiguous results, but they will help to establish public policies and political programs tailored to an existing reality.  I draw your attention to the following parts of the text: “Seven dimensions were identified through content analysis by applying a manual coding-approximation process, from which the general description and, subsequently, the scope of each dimension were obtained as outlined below. 3) A frequency count in Google Scholar of all definitions was performed, measuring the impact through citations to then calculate the relative use of each dimension. It is necessary to understand that the relationship of the identified constructs is not of equal significance, the most widely used having a greater impact ( Donahue et al., 2015; Howes & Solomon, 1951; Kageura & Umino, 1996; Luque & Herrero-García, 2019; Murphy, 1992).” and “This was achieved through the following procedure: a) The basic ideas to be used in the structuring of informality processes were defined, which in turn would serve to guide the research design; b) Once all of the definitions and approximations had been read, their depth and implications were observed and noted ( Bulmer, 1979; Nosofsky, 1986); c) From there, a theoretical framework was built for each dimension, justified by their treatment in the existing academic literature: in this case, seven were required. The same systematization can be replicated for other research, adapting the number of dimensions in relation to the existing literature that justifies them; d) Each definition of informality was analyzed at a conceptual level to discover whether it contained the preconfigured implications in each one of the dimensions; if so, they were included in Table 8 for subsequent counting and analysis.”

 The results of Tables are now better explained.

 The limits are better explained.

 As requested, a paragraph is included in footnote Nº2 to make reference to much of the existing legislation of the relevant supranational organizations. These are essential texts for the understanding of the progress and weaknesses in the informal economy processes.

# Reviewer report: Reviewer response for version 1

**DOI:** 10.21956/openreseurope.15076.r27992

**Published:** 2021-11-05

**Authors:** Fahri Fahri Özsungur

**Affiliations:** 1 Department of Labor Economics and Industrial Relations, Mersin University, Mersin, Turkey

In the study, phenomenology, research question, and method were put forward correctly. The flow of the work is well structured. The study is based on a conceptual analysis with the specific aim of clarifying whether informality is concerned only with economic aspects or whether conditions of greater depth or interconnectedness are involved. The subject tries to clarify an important and current phenomenon.

 There are guiding legal regulations of the European Union and the European Economic Community regarding the legality and necessity in the informal economy. By drawing a general framework, brief information about the sample practices and guidelines of the European Union on this subject can be added to the article.

 The reasons why the political dimension in the results included the least interaction with other categories should be discussed. The author's recommendations regarding the derivation of the meta-concept of the informal economy should be explained. What could be the underlying reason why the cultural dimension was the most neglected dimension in academic publications? Also, a "discussion" section should be included in the article. In this section, the cause-effect relationships of the findings should be discussed. In particular, the discussion of the causes and consequences of the political and labor categories vectorize the processes of informality only residually should take place in this section.

 The limitations of the method should be added to the article. In addition, all the limitations of the study should be included under the "Limitations" heading.  

 More information is needed on the application areas, possible consequences, and contributions of the proposed tetrad model. On the other hand, the theoretical framework of the article and the source of inspiration for the researched phenomenon should be explained in this section after the conceptual framework. Finally, the applicable recommendations regarding the development and informality processes of the model put forward with the findings should be discussed in the context of policy development.

If applicable, is the statistical analysis and its interpretation appropriate?

Yes

Is the study design appropriate and is the work technically sound?

Yes

Is the work clearly and accurately presented and does it engage with the current literature?

Yes

Are the conclusions drawn adequately supported by the results?

Yes

Are sufficient details of methods and analysis provided to allow replication by others?

Yes

Are all the source data and materials underlying the results available?

Yes

Reviewer Expertise:

Business, law, finance, social work, social policy, women studies

I confirm that I have read this submission and believe that I have an appropriate level of expertise to confirm that it is of an acceptable scientific standard, however I have significant reservations, as outlined above.

# Response

**Published:** 2021-12-08

**Authors:** Arturo Luque

**Affiliations:** Universidad Técnica de Manabí, Ecuador

Dear Dr. Fahri Fahri Özsungur (https://open-research-europe.ec.europa.eu/articles/1-134)

 First of all, I wish to thank you for the thoroughness, kindness and courtesy you have shown in your review.  I fully understand and value the work of a reviewer, which in many cases receives no other reward than the professional satisfaction of carrying out a vital task and the respect of fellow academics who, after months (or years) of work on a project, submit their papers for evaluation. As the lead author of this manuscript, I too review a number of articles throughout the year. In this case, your indications and advice seem entirely constructive, with the clear purpose of improving the study. Understandably, we have had to balance your comments with some of the recommendations of the other three reviewers in order to avoid contradictions in the text. I enjoy the challenge of incorporating corrections and suggestions and the process, of course, helps all writers to grow, learn and avoid future mistakes. Even where entire passages have needed to be altered in accordance with your review, I have diligently attempted to respond to your recommendations as enumerated below:

 The following observations have been taken into consideration and duly corrected: *“One of the observations is that in the Economic dimension the search chain: “weakening of control of regulatory bodies” would seem to deal with more legal aspects as they are control bodies. Similarly, the search string 'promotion and conservation of ancestral traditions' should be treated as a cultural rather than a political aspect.”*

 According to reviewer 2, *“It should be clarified in the methodology section that the search engine used was Google Scholar from the beginning”*, but this point is already clarified in the methodology: “A search was carried out for definitions of the informal economy and related constructs through the existing literature using indexed databases, which in turn were part of Google Scholar ( https://scholar.google.com/)”, and “The selection of the indexed academic databases to be used were: Web of Science, Scopus and Latindex with no time limit on date of publication or type of paper. Both English language and Spanish publications were included in the search between 12/09/2020 and 24/11/2020. 102 definitions were found by this method between 12/09/2020 and 24/11/2020”.The definitions were searched in three databases (Web of Science, Scopus and Latindex) and once they were found it was verified that they were also indexed in Google Scholar.  Consequently, we saw no need to provide further clarification.  

 For the compilation of Table Nº8, the dimensions and methodology were previously established and justified. This was done by drawing on high-impact articles that validate the method as well as finding justifications in the existing specific literature. Definitions as such are not interpreted but rather categorized in relation to dimensions. There is no doubt that, as the study points out, only an approximation is possible to many of the current trends in informal economy processes. These are not unambiguous results, but they will help to establish public policies and political programs tailored to an existing reality.  I draw your attention to the following parts of the text: “Seven dimensions were identified through content analysis by applying a manual coding-approximation process, from which the general description and, subsequently, the scope of each dimension were obtained as outlined below. 3) A frequency count in Google Scholar of all definitions was performed, measuring the impact through citations to then calculate the relative use of each dimension. It is necessary to understand that the relationship of the identified constructs is not of equal significance, the most widely used having a greater impact ( Donahue et al., 2015; Howes & Solomon, 1951; Kageura & Umino, 1996; Luque & Herrero-García, 2019; Murphy, 1992).” and “This was achieved through the following procedure: a) The basic ideas to be used in the structuring of informality processes were defined, which in turn would serve to guide the research design; b) Once all of the definitions and approximations had been read, their depth and implications were observed and noted ( Bulmer, 1979; Nosofsky, 1986); c) From there, a theoretical framework was built for each dimension, justified by their treatment in the existing academic literature: in this case, seven were required. The same systematization can be replicated for other research, adapting the number of dimensions in relation to the existing literature that justifies them; d) Each definition of informality was analyzed at a conceptual level to discover whether it contained the preconfigured implications in each one of the dimensions; if so, they were included in Table 8 for subsequent counting and analysis.”

 The results of Tables are now better explained.

 The limits are better explained.

 As requested, a paragraph is included in footnote Nº2 to make reference to much of the existing legislation of the relevant supranational organizations. These are essential texts for the understanding of the progress and weaknesses in the informal economy processes.
